# Integrating 16S rRNA Sequencing and LC–MS-Based Metabolomics to Evaluate the Effects of Live Yeast on Rumen Function in Beef Cattle

**DOI:** 10.3390/ani9010028

**Published:** 2019-01-19

**Authors:** Ibukun Ogunade, Hank Schweickart, Megan McCoun, Kyle Cannon, Christina McManus

**Affiliations:** College of Agriculture, Communities, and the Environment, Kentucky State University, Frankfort, KY 40601, USA; hank.schweickart@kysu.edu (H.S.); megan.mccoun@kysu.edu (M.M.); ogunadeibukun@gmail.com (K.C.); christina.mcmanus@kysu.edu (C.M.)

**Keywords:** bacterial diversity, beef steer, live yeast, ruminal metabolome

## Abstract

**Simple Summary:**

Live yeast products are used in ruminant nutrition to improve feed efficiency and performance. The effects of yeast on animal performance are mediated via alteration in the rumen microbial population and fermentation; however, the types of metabolites produced from feeding yeast additives have not been described. This study integrated 16S rRNA sequencing and LC–MS-based metabolomics to evaluate rumen bacterial diversity and metabolome of beef steers fed no or 15 g/d of live yeast product. Our findings confirm that live yeast supports the growth of fiber digesters, optimizes the utilization of oxygen and lactic acid, and inhibits the growth of pathogenic *Salmonella* in the rumen. In addition, some bacteria with unknown functions in relation to the effects of live yeast showed positive correlations with metabolites involved in the metabolism of amino acid and energy substrates. This study enhances our understanding of the effects of live yeast in the rumen.

**Abstract:**

We evaluated the effects of live yeast on ruminal bacterial diversity and metabolome of beef steer. Eight rumen-cannulated Holstein steers were assigned randomly to one of two treatment sequences in a study with two 25-d experimental periods and a crossover design. The steers were housed in individual pens. The dietary treatments were control (CON) or yeast (YEA; CON plus 15 g/d of live yeast product). Bacterial diversity was examined by sequencing the V3-V4 region of 16S rRNA gene. The metabolome analysis was performed using a liquid chromatograph and a mass spectrometry system (LC–MS). Live yeast supplementation increased the relative abundance of eight cellulolytic bacterial genera as well as *Anaerovorax* and *Lachnospiraceae*. *Proteiniclasticum*, *Salmonella*, and *Lactococcus* were not detected in the YEA treatment. Live yeast supplementation increased the concentrations of 4-cyclohexanedione and glucopyranoside and decreased the concentrations of threonic acid, xanthosine, deoxycholic acid, lauroylcarnitine, methoxybenzoic acid, and pentadecylbenzoic acid. The *bacteroidales* BS11, *Christensenellaceae* R-7, and *Candidatus saccharimonas* showed positive correlations with the metabolites involved in amino acid biosynthesis and the metabolism of energy substrates; the functions of these bacteria are not fully understood in relation to the mode of action of yeast. This study confirms the usefulness of LC–MS-based metabolomics in deciphering the mode of action of live yeast in the rumen.

## 1. Introduction

Live yeast products containing *Saccharomyces cerevisiae* are used as a feed additive in ruminant nutrition to improve feed efficiency and performance and prevent health disorders, such as ruminal acidosis [1]. Yeast products have a number of positive effects on the rumen environment and microbial activities [2,3], including the improved growth and activity of fiber-degrading microorganisms in the rumen [4]. Yeast also has the potential to reduce dietary energy loss in the rumen [5]. The cells of *S. cerevisiae* can provide nutrients, such as B vitamins and amino acids, for rumen microbes [6]. Yeast can also scavenge oxygen, thereby creating a more anaerobic environment [7]. Although the effects of yeast on animal performance seem to be mediated via the effects on rumen fermentation, the biggest challenge with viable yeast supplementation is the lack of an established mode of action. It is possible that the rumen fermentation variables measured, such as pH, fermentation acids, and the microbial population, explain only a fraction of the response. Next-generation high-throughput sequencing (via 16S sequencing) has been used to elucidate the effects of yeast products on the rumen microbial population [8]. However, alterations in the types of metabolites produced as a result of feeding yeast additives have not been completely described. Recent studies have applied metabolomics, the comprehensive analysis of all metabolites in a biological system, to predict feed efficiency [9,10], evaluate dietary responses to different feeds [11], assess the milk quality of ruminants [12], and evaluate the effect of monensin on the rumen microbial population [13]. However, no studies have applied metabolomics to study the effects of live yeast additives. This study integrated 16S ribosomal ribonucleic acid (rRNA) sequencing and liquid chromatography–mass spectrometry (LC–MS)-based metabolomics to evaluate the effects of a live yeast product on ruminal bacterial diversity and metabolome in the beef steer.

## 2. Materials and Methods 

All experimental animals were managed according to the guidelines approved by the Institutional Animal Care and Use Committee of Kentucky State University (protocol number 18-001).

### 2.1. Animals, Housing, and Feeding

Eight rumen-cannulated Holstein steers (mean ± SD body weight: 504 ± 45 kg) were assigned randomly to 1 of 2 treatment sequences in a study with two 25-d experimental periods and a crossover design. The steers were housed in individual pens and were fed 50% each of concentrate-mix and red clover/orchard hay ad libitum. Mineral mix (Hubbard Feeds; Mankato, MN, USA) was fed free choice. The dietary treatments were (1) control (CON; basal diet without additive) and (2) yeast (YEA; basal diet plus 15 g/d of Peloton live yeast feed additive; PMI, Arden Hills, MN, USA). Dietary ingredients and chemical compositions are shown in Table 1. The yeast additive was top-dressed on the concentrate mix from day 1 to 25 of each period for only the YEA treatment. A 10-day washout period was imposed between the two periods to minimize the carryover of treatment effect.

### 2.2. Rumen Fluid Sampling

Representative samples (200 mL) of the ruminal contents were collected via the cannula by spot sampling from different parts of the rumen at 3, 6, and 9 h after the morning feeding on day 25 of each period. The ruminal contents were hand-strained through 4 layers of cheesecloth to separate solid and liquid fractions. The samples of the solid and liquid phases for each collection day were composited, mixed 1:1 (*w/w*), and stored at −80 °C until further analysis.

### 2.3. DNA Extraction, Sequencing, and Diversity Analysis 

Total DNA was extracted from the rumen contents with a PowerSoil DNA Isolation Kit which is effective at removing PCR inhibitors (MOBIO Laboratories Inc.; Carlsbad, CA, USA). The integrity of the DNA was verified by agarose (0.7%) gel electrophoresis. The DNA samples were prepared for sequencing according to the Illumina 16S Metagenomic Sequencing Library protocols to amplify the V3–V4 region. The DNA quality was measured by PicoGreen and Nanodrop (Thermo Fisher Scientific Inc., Eugene, OR, USA). An amount of 10 ng of genomic DNA was amplified using the following polymerase chain reaction (PCR) conditions: 94 °C for 3 min followed by 35 cycles of 94 °C for 15 s, 55 °C for 45 s, and 72 °C for 1 min, followed by a final elongation step of 8 min at 72 °C. The barcoded fusion primer sequences used for amplifications were 519F:5′-CCTACGGGNGGCWGCAG-3′ and 806R: 5′-GACTACHVGGGTATCTAATCC-3′. The final purified product was quantified using real-time quantitative PCR according to the qPCR Quantification Protocol Guide (KAPA Library Quantification kits for Illumina Sequencing platforms, Wilminton, MA, USA) and qualified using the LabChip GX HT DNA High Sensitivity Kit (PerkinElmer; Waltham, MA, USA). Amplicons were performed on a paired-end Illumina HiSeq2500 platform to generate 250-bp paired-end raw reads. The paired-end reads were merged using fast length adjustment of short reads [14]. The resulting raw tags were quality-filtered using specific filtering conditions of Trimmomatic v0.33 quality control process (http://www.usadellab.org/cms/?page=trimmomatic; [15]). The resulting clean tags were compared with the reference database (the “Gold” database, http://drive5.com/uchime/uchime_download.html; [16]) using the UCHIME algorithm [17] to detect and remove the chimeric sequences. We used UCLUST [16] in QIIME (version 1.8.0) to cluster the tags with 97% similarity and acquired the Operational Taxonomic Units (OTUs). The representative OTU sequences were aligned with the rRNA database (Silva) for taxonomic assignment. The OTUs, which were annotated as mitochondria, chloroplasts, and unknown, were removed. Alpha diversity (Shannon index) and beta diversity indices, based on unweighted unifrac and weighted unifrac distances, were generated using the QIIME software package with a script core_diversity_analyses.py [18].

### 2.4. Statistical Analysis

Variables such as the relative abundance of bacteria, number of reads, and diversity indices were analyzed using the GLIMMIX procedure of SAS version 9.4 (SAS Institute Inc., Cary, NC, USA) and a model that included the effects of the treatment, period, and their interaction. The differences between means were determined using the Fisher’s test. Significant differences were declared at *p* ≤ 0.05. Normality was tested by examining the distribution of residuals. Linear discriminant analysis effect size (LEfSe) which performs a Kruskal–Wallis (KW) test followed by linear discriminant analysis, was used to identify the most differentially abundant taxa [19]. The significance estimate for the Kruskal–Wallis (KW) test and the logarithmic linear discriminant analysis (LDA) score cutoff were 0.05 and 4.0, respectively.

### 2.5. Non-Targeted Metabolomics Analysis

#### 2.5.1. Sample Preparation and Analysis

The samples were prepared according to procedures described by Ogunade et al. [13]. Briefly, 500 µL of the ruminal fluid sample was mixed with 2 mL of methanol–water (1:1, *v*/*v*) and then vortex-mixed for 2 min. The mixture was then centrifuged at 15,000 × g for 10 min at 4 °C. The resulting supernatant was dried in a vacuum concentrator, and then suspended in 200 µL methanol/water mixture (1:1 vol/vol).

The analysis was performed on an UltiMateTM 3000 ultra-performance liquid-chromatography (UPLC) system (Thermo Fisher Scientific, Waltham, MA, USA) with a Waters Atlantis T-3 column (100 mm × 2.1 mm; 1.8-µm particle size) at 35 °C and an injection volume of 5 µL. The UPLC system was equipped with an autosampler and coupled with an Orbitrap Velos mass spectrometer (MS). The mobile phases (flow rate of 0.5 mL/min) consisted of 0.1% formic acid (*v*/*v*) in double-distilled water (eluent A) and 0.1% formic acid (*v*/*v*) in acetonitrile (eluent B). The gradient elution used was: 0–9 min, 100%–0% A; 9–12 min, 100% A; 12–15 min, 100–0% A. Mass spectrometry was done in positive and negative modes (spray voltage = 3.5 kV). The capillary and source-heat temperature was set at 350 °C, and the respective flows for sheath and auxiliary gas were 40 and 10 L h^−1^. The quality control samples were analyzed every 4 samples to validate the stability and repeatability of the UPLC/MS system.

The Ammonia-N concentration of the rumen fluid samples was measured after centrifuging at 10,000 × g for 15 min at 4 °C using the phenol–hypochlorite technique [20]. Volatile fatty acids (acetate, propionate, and butyrate) and lactate were quantified using a High-Performance Liquid Chromatograph system (Hitachi L2400, Tokyo, Japan) fitted with an Aminex HPX-87H column (Bio-Rad Laboratories, Hercules, CA, USA). The mobile phase was a 0.015 M sulfuric acid at a flow rate of 0.7 mL/min at 50 °C [21].

#### 2.5.2. Data and Statistical Analysis 

The raw data were converted to Analysis Base File format by Reifycs ABF Converter (http://www.reifycs.com/AbfConverter/index.html). Individual chromatographic peaks were identified based on retention time and mass to charge ratio (*m*/*z*) values using MS-DIAL version 2.84 [22]. Identified metabolites were then merged and imported into MetaboAnalyst 4.0 [23]. The data were first log-transformed and pareto-scaled. Principal Components Analysis (PCA) was then used for data visualization and outlier detection. Orthogonal partial least squares discriminant analysis (OPLS–DA), a tool used for dimension reduction and identification of spectral features that drive group separation [24], was used to identify the differential metabolites. The identified differential metabolites were filtered using significance estimate of *p* ≤ 0.10 and fold change (FC > 1.2) of the peak intensities (mean value of peak intensity obtained from YEA group/mean value of peak intensity obtained from CON group). Pearson correlation analysis was used to examine the association between the relative abundance of dominant ruminal bacteria (>1%) that were affected by yeast supplementation and peak intensities of the rumen metabolites. The Pearson correlation coefficients were generated using R software (http://www.r-project.org) and were declared significant at *p* ≤ 0.05.

## 3. Results

### 3.1. Sequencing Results 

Quality filtering and the removal of chimeric sequences yielded 1,027,901 sequences with a mean sequence length of 419 bases and an average coverage of 64,243 sequences per sample. Rarefaction analysis and average Good’s coverage of 0.99 ± 0.002 for all samples showed that the number of sequences used for the analysis was sufficient to determine the total number of sequence types (Figure S1).

### 3.2. Diversity and Relative Abundance of Taxa 

**Figure 1:**
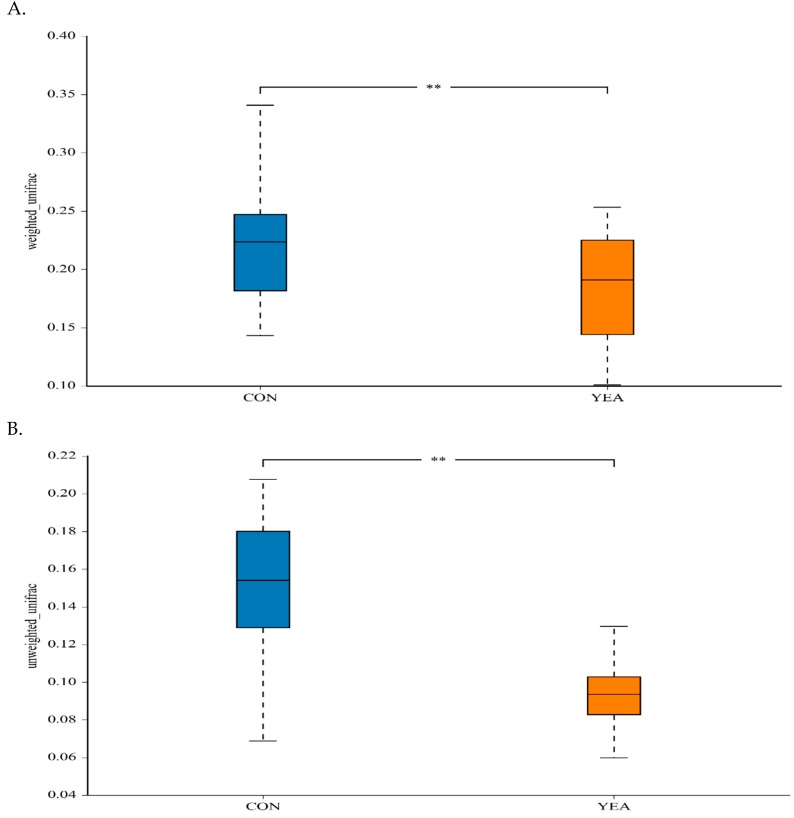
(**A**) Between-sample (β) diversity indices for the weighted and (**B**) unweighted uniFrac distances of rumen samples from beef steers fed no (control; CON) or 15 g/d of live yeast product (YEA; PMI, Arden Hills, MN, USA), ** *p* < 0.05.

The treatment did not affect the Shannon index, a measure of within-sample (α) phylogenetic diversity that gives the measurement of both species number and the distribution of the abundance (Figure S2). The addition of YEA to the diet reduced the between-treatment diversity estimated by weighted and unweighted uniFrac distances (Figure 1).

At the phylum level, *Firmicutes* (49.6 ± 6.57%) dominated the bacterial community, followed by *Bacteroidetes* (37.4 ± 6.11%), *Proteobacteria* (4.92 ± 7.56%), and then *Saccharibacteria* (4.58 ± 2.24%; Figure S3). The relative abundance of *Saccharibacteria* (5.81 vs. 3.35%) was increased (*p* = 0.03) by the YEA diet. At the genus level, *Prevotella* dominated (12.7 ± 4.87%) the bacterial community, followed by *Rikenellaceae* RC9 gut group (11.4 ± 2.38%), *Christensenellaceae* R-7 group (6.04 ± 2.51%), and then an uncultured rumen bacterium belonging to the *Bacteroidales* BS11 gut group (5.75 ± 2.83%; Figure S4). Dietary treatment affected 44 genera (*p* ≤ 0.05; Table S1). The affected genera with relative abundance of at least 0.1% are shown in Table 2.

**Figure 2:**
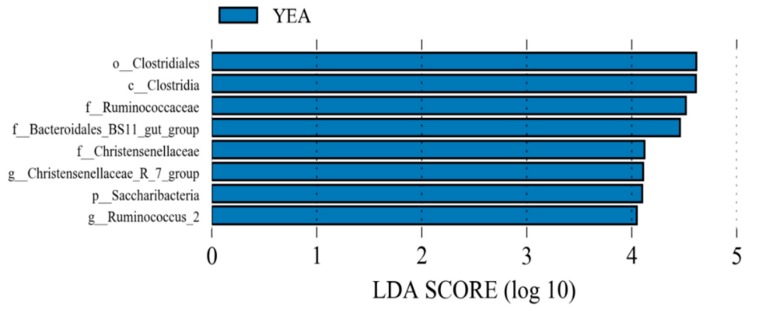
Linear discriminant analysis effect size of rumen bacterial populations of beef steer fed no (control) or 15 g/d of live yeast product (YEA; PMI, Arden Hills, MN, USA). This plot indicates the most differentially abundant taxa according to the logarithmic linear discriminant analysis (LDA) score cutoff of ≥4.0. All the taxa meeting the significant threshold of 4.0 are enriched in steers fed YEA.

Dietary yeast supplementation increased (*p* ≤ 0.05) the relative abundance of *Ruminococcaceae* NK4A214 group, *Christensenellaceae* R-7 group, *Ruminococcaceae* UCG-010, *Candidatus Saccharimonas*, uncultured bacterium (*Bacteroidales* BS11 gut group), *Ruminococcus* 2, *Anaerovorax*, *Lachnospiraceae* UCG-008, and *Ruminococcaceae* UCG-005, while those of *Lachnoclostridium*, *Lachnoclostridium* 5, and *Bacillus* were reduced (*p* ≤ 0.05). Using LEfSe, the YEA diet increased the relative proportion of *Saccharibacteria* at the phylum level and *Christensenellaceae* R-7 group and *Ruminococcus* 2 at the genus level (Figure 2).

A total of 962 OTUs were shared between the two treatments whereas 14 OTUs were unique to CON and five OTUs were unique to YEA. Genera such as *Arcticibacter, Comamonas, Mobilitalea, Mogibacterium, Morganella, Proteiniclasticum, Salmonella, Serratia, Clostridium_sensu_stricto 7, Lachnoclostridium, Acinetobacter, Sphaerochaeta, Lactococcus,* and *Tyzzerella* were not detected in the YEA treatment. Uncultured bacterial species belonging to the family *Bacteroidales* BS11 gut group and genera *Rikenellaceae* RC9 gut group and *Prevotellaceae* UCG-003 were unique for YEA.

### 3.3. Rumen Fluid Metabolome

**Figure 3:**
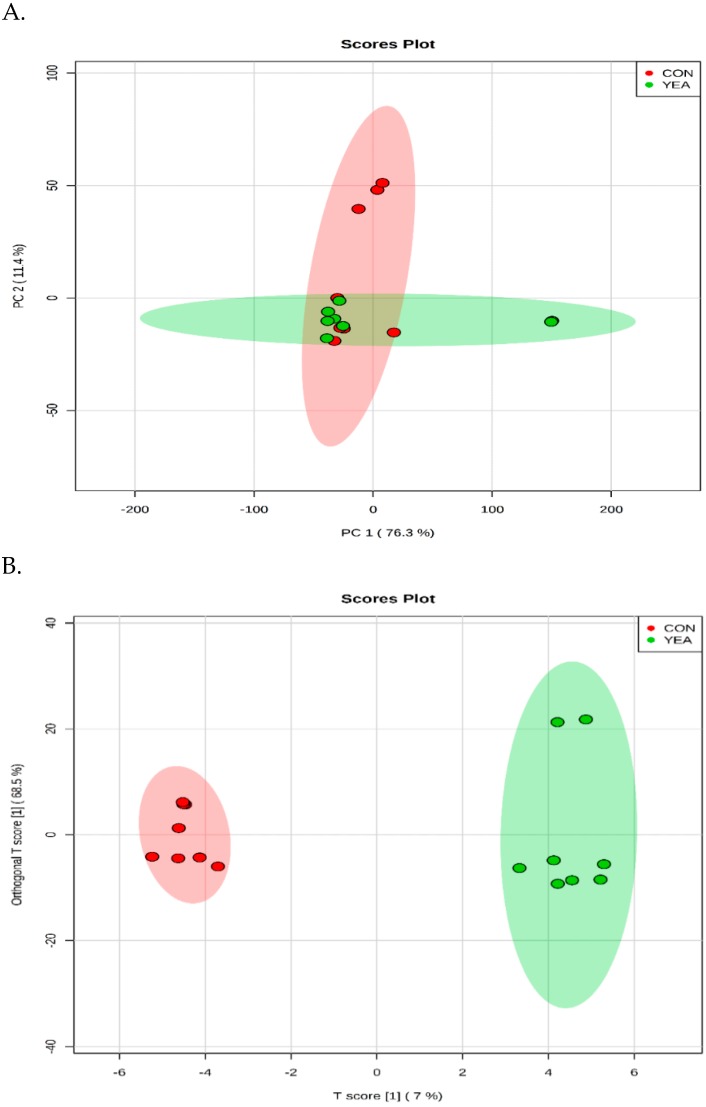
(**A**) The scores plot of the PCA model showing the directions that best explain the variance between the two treatments. (**B**) OPLS–DA score plot of all metabolite features. CON = steers fed control diet (no live yeast product), YEA = steers fed 15 g/d of live yeast product (PMI, Arden Hills, MN, USA). Each data point represents one rumen fluid sample.

A total of 311 metabolites were identified (Table S2). The PCA and OPLS–DA score plot revealed a good separation between the CON and YEA treatments (Figure 3). The *p*-Value for 100 permutations was 0.02, which confirms the validity of the OPLS–DA model (R^2^ = 0.99, Q^2^ = 0.48). 

Eight metabolites were differentially expressed (*p* ≤ 0.10; Table 3). 4-cyclohexanedione and methyl β-d-glucopyranoside were increased, whereas threonic acid, xanthosine, deoxycholic acid, lauroylcarnitine, methoxybenzoic acid, and 2-acetoxy-6-pentadecylbenzoic acid were decreased by the YEA diet relative to CON.

Among the dominant bacteria that responded to yeast supplementation, the relative abundance of *Candidatus saccharimonas* was positively (r ≥ 0.5; *p* ≤ 0.05) correlated with three metabolites (spermidine, quinolone, and thiacloprid). The relative abundance of the *Christensenellaceae* R-7 group was positively correlated (r ≥ 0.5; *p* ≤ 0.05) with 28 metabolites whereas the uncultured bacterium in *Bacteroidales* BS11 gut group was positively correlated (r ≥ 0.5; *p* ≤ 0.05) with 27 metabolites (Table S3). Table 4 shows those metabolites that are involved in one or more metabolic pathways.

Acetate was greater (*p* = 0.01; 57.9 vs. 54.6 mM) and ammonia-N was lower (*p* = 0.01) in steers receiving the YEA diet than those receiving the CON diet (3.07 vs. 3.87 mM; Table 5).

## 4. Discussion

Numerous studies have evaluated the effects of yeast products containing live *Saccharomyces cerevisiae* on the rumen microbial population of ruminants via 16S gene sequencing [25]. However, to date, no studies have been published that integrate 16S rRNA sequencing and LC–MS-based metabolomics to evaluate the effect of live yeast cultures on rumen microbial activities and function. It is important to note that the comparisons among trials of the responses to yeast supplementation require clarification and caution because of the diversity of yeast products, their composition, processing, and the yeast cell wall components [26]. The product used in this study contains a thermally stable blend of live *Saccharomyces cerevisiae* yeast and a yeast cell wall product. 

The fact that the YEA diet increased the relative abundance of cellulolytic bacteria, such as *Ruminococcaceae* NK4A214 group, *Ruminococcaceae UCG-010, Ruminococcaceae UCG-005, Candidatus Saccharimonas,* and *Ruminococcus* 2, is consistent with previous studies demonstrating that some strains of live *S. cerevisiae* favor the establishment of cellulolytic bacteria in the rumen [27]. Consequently, yeast supplementation increases fiber digestibility [28,29,30], dry matter intake, and the performance of ruminants [31,32,33]. Tong et al. [34] compared the ruminal microbiota in high-yielding and low-yielding dairy cows and observed higher relative abundance of *Ruminococcus 2*, *Lachnospiraceae*, *Christensenellaceae*, and *Ruminococcaceae* NK4A214 in high-yielding cows. The fact that these bacteria were increased by the YEA diet partly explains the increased feed efficiency of beef and dairy cattle fed a diet supplemented with *S. cerevisiae* [35,36].

Some species of *Bacillus* have been tested as direct-fed microbial (DFM) supplements for ruminants because of their strong cellulolytic activity and mechanisms to inhibit gastrointestinal infection [37]. *Bacillus* spp. in the rumen can produce polysaccharidases and glycoside hydrolases to utilize polysaccharides [38]. The addition of *B. licheniformis* and *B. subtilis* to diets reduced the mortality of young lambs and increased the performance of ewes [39]. Another study showed that supplementation of bacilli DFM containing *B. licheniformis* increased the total volatile fatty acid and acetate concentrations in Holstein cows [40]. The fact that the YEA diet reduced the relative abundance of *Bacillus* in this study does not mean this additive is likely to reduce fiber digestion because *Bacillus* was less prevalent than other cellulolytic bacteria and these were increased with YEA supplementation.

*Salmonella* infection is a critical animal health and food safety issue [41]. *Salmonella* was not detected in steers fed the YEA diet in this study. Thus, our results support the anti-*Salmonella* effects of products containing *S. cerevisiae* found in other studies [42,43]. Magalhães et al. [42] observed improved gastrointestinal health in pre-weaned dairy calves naturally exposed to *Salmonella*. Similarly, Brewer et al. [43] observed reduced intestinal colonization and shedding of *Salmonella* in calves fed a *S. cerevisae* fermentation product. The mechanism of action is likely related to reduced colonization by pathogens in the gut because the cell wall of *S. cerevisiae* contains mannan oligosaccharide, which can act as a high-affinity ligand that binds gram-negative bacteria, such as *Salmonella* and *E. coli* that possess mannose-specific type-1 fimbriae [44,45].

The suggested effects of certain strains of yeast, such as the oxygen-scavenging function [46], a decreased ruminal lactate concentration [47,48], increased carbohydrate digestion [49], and the increased flow of duodenal amino acids [50], are supported by the findings that some of the OTUs that were unique to the steers fed the CON diet were aerobic (*Comamonas* and *Arcticibacter*), ruminal lactic acid producers (*Lactococcus*), asaccharolytic (*Mogibacterium*), and amino acid fermenters (*Proteiniclasticum*) [51,52,53,54]. The ability of some strains of *S. cerevisiae* to scavenge oxygen in the rumen from the surface of freshly ingested feeds may also explain the greater relative abundance of *Anerovorax*, a strictly anaerobic bacterium [55], in steers fed the YEA diet.

The relative abundance of an uncultured bacterium belonging to the *Bacteroidales* BS11 gut group was increased by the YEA diet. This bacterium was positively correlated with some metabolites that are involved in amino acid metabolism and biosynthesis, as well as the metabolism of energy sources, such as starch, sucrose, and galactose. This result is consistent with the lower ruminal ammonia concentrations observed in steers receiving yeast supplements in this study as well as other studies [56,57]. The decrease in rumen ammonia-N concentrations is likely due to an increase in the efficiency of microbial protein synthesis as more energy is supplied from the metabolism of starch and sucrose. Yeast supplements increase organic matter degradation rates and thereby improve the release of energy that can be used for microbial growth [58,59]. Similarly, the positive associations between the *Christensenellaceae* R-7 group and *Candidatus saccharimonas* with several metabolites involved in pathways associated with the metabolism of amino acids and energy indicate that these bacteria also play important roles in rumen fermentation. Also, some uncultured bacterial species belonging to family *Bacteroidales* BS11 gut group, and genera *Rikenellaceae* RC9 gut group and *Prevotellaceae* UCG-003 were unique for YEA. Future studies should aim to identify these species and examine the functions of the aforementioned bacteria to understand their roles in the mode of action of yeast.

The roles of most of the differential rumen metabolites detected in this study have not been fully described. Methyl β-d-glucopyranoside is a glycoside that is resistant to fermentation by *S. cerevisiae* [60]. Threonic acid is a primary oxidation product of the interaction of ascorbic acid and radical oxygen species [61]. The decreased levels of threonic acid in the rumen of steers fed the YEA diet probably indicate a decrease in levels of radical oxygen species, which supports the oxygen-scavenging function of *S. cerevisiae*. Xanthosine and ammonia are products of guanosine deamination, one of the major steps in purine catabolism to uric acid [62]. The decreased levels of xanthosine in steers fed the YEA diet probably indicate reduced deamination activity in the rumen, which supports the lower levels of ammonia-N observed in this study. Lauroylcarnitine is a long-chain fatty acid ester of carnitine with lauric acid whereas deoxycholic acid is a secondary bile acid that facilitates fat absorption and cholesterol excretion [63,64]. These aforementioned metabolites are important candidates for future studies because of their response to yeast supplementation.

Increased acetate level in beef steers fed the YEA diet reflects increased fiber digestibility because acetate is the primary product of cellulolytic bacteria [65]. Similar results were observed in steers fed a diet containing 50% forage and 50% concentrate and in lactating dairy cows fed a diet containing 43% forage and 57% concentrate [50,66]. Lactate was not influenced in this study, possibly because the level was very low due to the non-acidotic diet fed. The responses of rumen volatile fatty acids to yeast supplementation are influenced by dietary composition. The influence of yeast supplementation on rumen volatile fatty acids is greater when using high-concentrate or high fiber diets [32]. Yeast supplementation reduces ruminal lactate accumulation in ruminants fed a high-concentration diet by increasing the activities of lactate-utilizing bacteria and/or decreasing the activity of lactate-producing bacteria, and/or favoring the conversion of lactate to propionate [67,68]. In high fiber diets, yeast increases rumen cellulolysis by improving the activities of cellulolytic bacteria [32].

The use of 16S rRNA sequencing in this study is a major limitation because it offers limited taxonomical and functional resolution [69]. In general, OTUs are less precise at the species level [70]. Future studies are needed to confirm the results of this study using shotgun metagenomics that provides a more precise taxonomic and functional classification of sequences [69]. Another limitation is that MS-based non-targeted metabolomics relies on comparing peak intensity values for evaluating the relative abundance of metabolites, which often lacks accuracy [71]. In addition, it is difficult to accurately identify metabolites due to the chemical diversity of the metabolome [72]. Despite this limitation, the results of this study suggest the usefulness of LC–MS-based metabolomics in deciphering the mode of action of live yeast.

## 5. Conclusions

In conclusion, supplementation with YEA increased the relative abundance of some cellulolytic bacteria and other bacteria that have positive associations with metabolites involved in amino acid and energy metabolism, confirming the effects of YEA on increasing the activities of ruminal cellulolytic bacteria and improving the nutritional status of the animal. Despite the low number of differentially expressed metabolites, this study enhances our understanding of the effects of live yeast in the rumen. Further in-depth studies are warranted in this field. 

## Figures and Tables

**Table 1:** Chemical composition of the diet.

Item	Red Clover/Orchard Grass Hay Mixture	Concentrate Supplement ^1^
Dry matter (%)	92.6	89.3
Neutral detergent fiber (% DM)	58.9	45.3
Acid detergent fiber (% DM)	40.2	24.4
Crude protein (% DM)	11.4	14.3
Ether extract (% DM)	NA ^2^	2.44
Starch (% DM)	NA ^2^	23.6

^1^ Concentrate supplement contains corn gluten meal, soy hull, and cracked corn in equal proportions. ^2^ Not measured, guaranteed analysis of the mineral mix (Hubbard Feeds: Mankato, MN, USA) fed free choice; 8.0% calcium, 6% phosphorus, 14% magnesium, 12 ppm cobalt, 2000 ppm copper, 55 ppm iodine, 4800 ppm manganese, 36.4 ppm selenium, 4800 ppm zinc, 100,000 IU/lb vitamin A, 20,000 IU/lb vitamin D, and 250 IU/lb vitamin E.

**Table 2:** Relative abundance of the dominant ruminal bacterial genera (>0.1% of total sequences) of beef steers fed no or 15 g/d of live yeast product.

Item	Treatment ^1^		SEM	*p*-Value
	CON	YEA		
*Ruminococcaceae* NK4A214 group	3.27	4.99	0.40	0.01
*Candidatus_Saccharimonas*	3.35	5.81	0.61	0.01
*Christensenellaceae* R-7 group	4.80	7.29	0.68	0.03
*Bacteroidales BS11* gut group *	1.11	2.36	0.21	0.01
*Ruminococcaceae UCG-010*	0.67	1.00	0.16	0.01
*Ruminococcus 2*	1.53	4.01	0.93	0.03
*Anaerovorax*	0.44	0.72	0.08	0.01
*Lachnoclostridium*	0.22	0.00	0.09	0.04
*Lachnoclostridium 5*	0.35	0.02	0.11	0.04
*Lachnospiraceae UCG-008*	0.10	0.21	0.01	0.02
*Ruminococcaceae UCG-005*	0.21	0.30	0.02	0.02
*Bacillus*	0.22	0.00	0.09	0.03

^1^ CON = no yeast treatment; YEA = 15 g/d of live yeast fermentation product (PMI, Arden Hills, MN, USA). * Uncultured bacteria belonging to *Bacteroidales BS11* gut group.

**Table 3:** Fold changes of differential ruminal metabolites of beef steers fed no or 15 g/d of live yeast product.

Metabolites	RT ^1^	FC ^2^	*p*-Value
4-cyclohexenedione	8.76	1.21	0.01
Methoxybenzoic acid	6.02	0.62	0.03
Threonic acid	0.61	0.38	0.03
2-acetoxy-6-pentadecylbenzoic acid	7.06	0.32	0.05
Methyl β-d-glucopyranoside	8.76	1.26	0.07
Lauroylcarnitine	8.13	0.55	0.07
Xanthosine	2.05	0.14	0.08
Deoxycholic acid	7.01	0.38	0.09

^1^ RT = retention time. ^2^ FC = fold change; the mean value of peak intensity, obtained from the yeast group/mean value of peak intensity which was obtained from the control group. FC values >1 mean that the metabolite is greater in steers fed yeast and FC values <1 mean that the metabolite is lower in steers fed yeast.

**Table 4:** List of metabolites associated with dominant rumen bacteria (>1%) affected by live yeast supplementation and their associated metabolic pathways.

Metabolites	Metabolic Pathway
***Christensenellaceae* R-7 group**	
Hypoxanthine	Purine metabolism
Hydroquinone	Riboflavin metabolism
Guanine	Purine metabolism
Glucose-1-phosphate	Glycolysis or gluconeogenesis, pentose and glucuronate interconversions, starch and sucrose metabolism, galactose metabolism, amino sugar and nucleotide sugar metabolism
Citrulline	Arginine and proline metabolism
Choline	Glycerophospholipid metabolism, glycine, serine and threonine metabolism,
5-hydroxyindole-3-acetic acid	Tryptophan metabolism
**Uncultured bacterium (*Bacteroidales* BS11 gut group)**	
Glucose-1-phosphate	Glycolysis or gluconeogenesis, pentose and glucuronate interconversions, starch and sucrose metabolism, galactose metabolism, amino sugar and nucleotide sugar metabolism
Citrulline	Arginine and proline metabolism
Choline	glycerophospholipid metabolism, glycine, serine and threonine metabolism
Alanine-valine	Aminoacyl-tRNA biosynthesis, valine, leucine and isoleucine biosynthesis, selenoamino acid metabolism, alanine, aspartate and glutamate metabolism
***Candidatus Saccharimonas***	
Spermidine	Beta-alanine metabolism, glutathione metabolism, arginine and proline metabolism

**Table 5:** Rumen fermentation of beef steers fed no or 15 g/d of live yeast product.

Item	Treatment ^1^		SEM	*p*-Value
	CON	YEA		
Acetate (mM)	54.6	57.9	1.09	0.01
Propionate (mM)	24.9	26.5	0.81	0.18
Butyrate (mM)	11.2	12.5	0.68	0.36
Lactate (mM)	1.16	0.94	0.51	0.67
Ammonia-N (mM)	3.87	3.07	0.16	0.01

^1^ CON = no yeast treatment; YEA = 15 g/d of live yeast fermentation product.

## Supplementary Materials

The following are available online at http://www.mdpi.com/2076-2615/9/1/28/s1, Figure S1: Rarefaction curve of the sequences, Figure S2: Within-sample phylogenetic diversity, Figure S3: Relative abundance of bacterial phyla, Figure S4: Relative abundance of the bacterial genera, Table S1: Treatment effects on the relative abundance of bacterial genera, Table S2: List of identified metabolites, Table S3: List of metabolites with positive correlations with rumen bacterial genera.
