# Supplementary material for: Integrating 16S rRNA Sequencing and LC–MS-Based Metabolomics to Evaluate the Effects of Live Yeast on Rumen Function in Beef Cattle
Source: Animals (Basel). 2019 Jan 19;9(1):28. doi: 10.3390/ani9010028 (PMC6356510; doi:10.3390/ani9010028)
Supplement: Supplementary file 1 [file animals-09-00028-s001.zip › animals-408936-Supplementary Figures S1-S4.docx]

Supplementary Materials: Integrating 16S rRNA Sequencing and LC–MS-Based Metabolomics to Evaluate the Effects of Live Yeast on Rumen Function in Beef Cattle

Ibukun Ogunade *, Hank Schweickart, Megan McCoun, Kyle Cannon and Christina McManus

College of Agriculture, Communities, and the Environment, Kentucky State University, Frankfort 40601 KY, USA; hank.schweickart@kysu.edu (H.S.); megan.mccoun@kysu.edu (M.M.); ogunadeibukun@gmail.com (K.C.); christina.mcmanus@kysu.edu (C.M.)

***** Correspondence: ibukun.ogunade@kysu.edu

Received: 30 November 2018; Accepted: 12 January 2019; Published: date


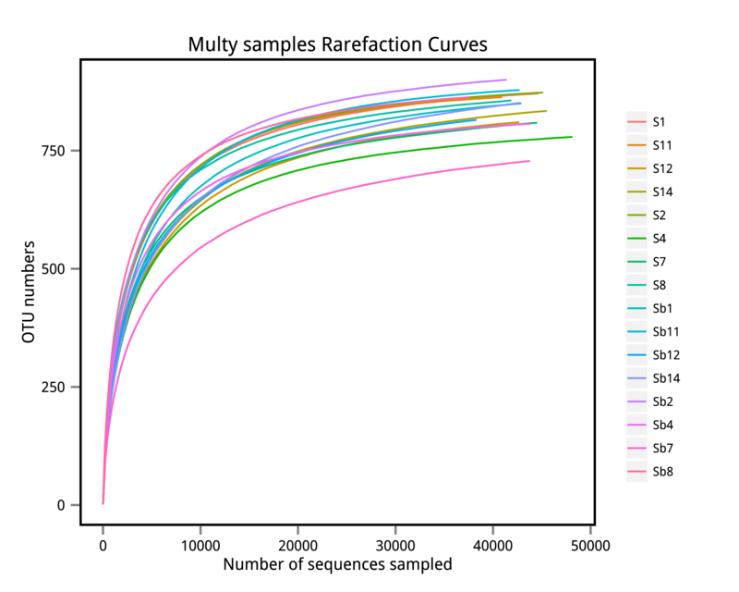


**Figure S1.** Rarefaction curve of the sequences.

**.**

**Figure S2.** Within-sample (α) phylogenetic diversity (*p* = 0.36; SE = 0.38). CON (control) = no yeast treatment; YEA = 15 g/d of live yeast product (PMI Nutritive Additives, Arden Hills, MN, USA).


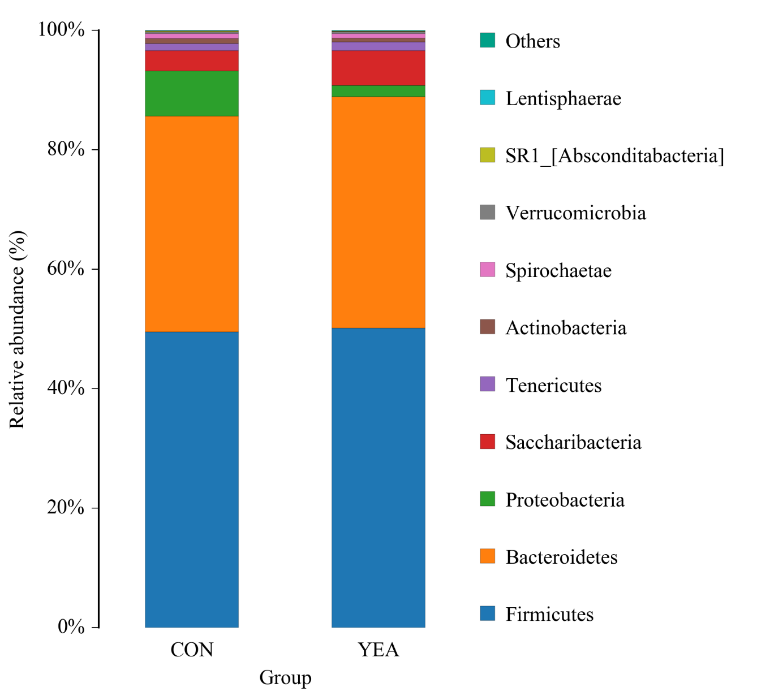
.

**Figure S3.** Relative abundance of bacterial phyla. CON (control) = no yeast treatment; YEA = 15 g/d of live yeast product (PMI Nutritive Additives, Arden Hills, MN, USA).

**
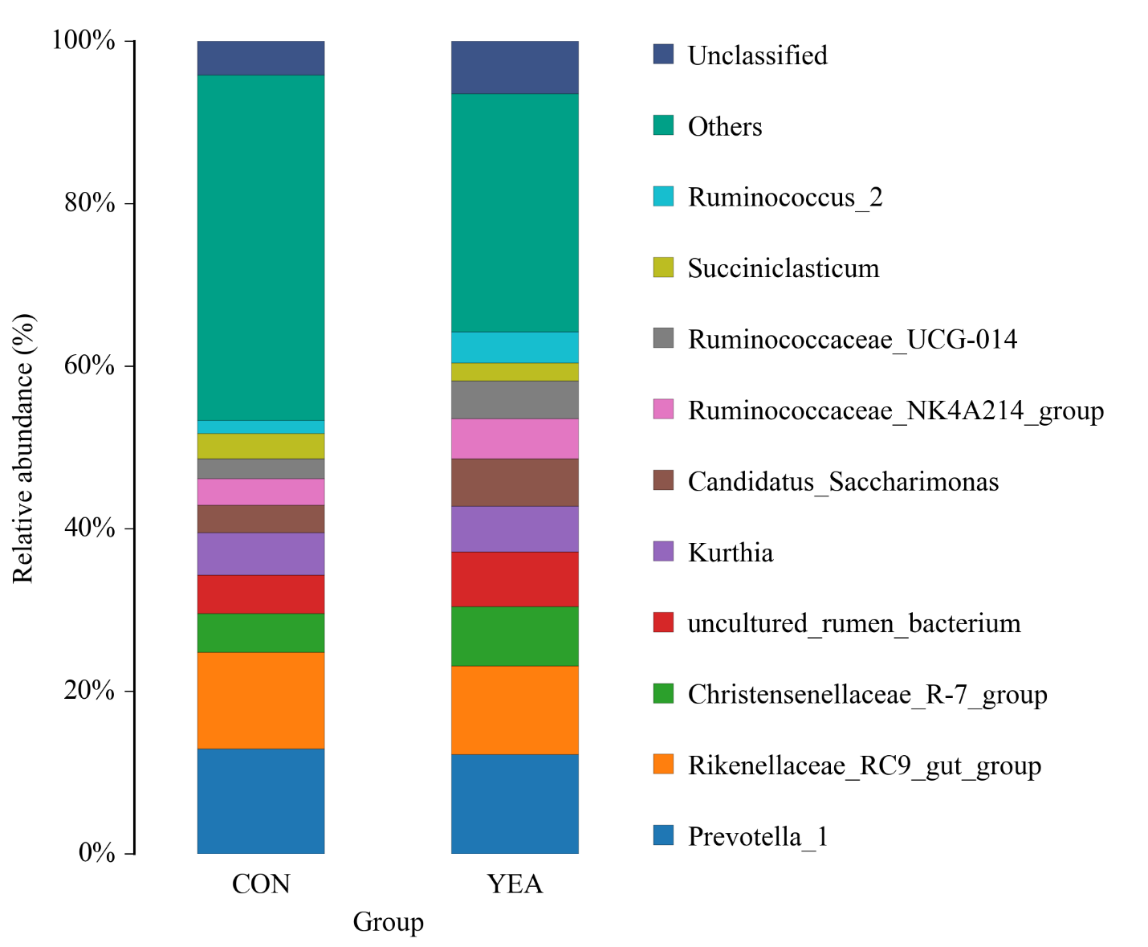
.**

**Figure S4.** Relative abundance of the bacterial genera. CON (control) = no yeast treatment; YEA = 15 g/d of live yeast product (PMI Nutritive Additives, Arden Hills, MN, USA).
